# SIRT3 regulates cardiolipin biosynthesis in pressure overload-induced cardiac remodeling by PPARγ-mediated mechanism

**DOI:** 10.1371/journal.pone.0301990

**Published:** 2024-04-16

**Authors:** Ling-Xin Liu, Xue-Hui Zheng, Jing-Han Hai, Chun-Mei Zhang, Yun Ti, Tong-Shuai Chen, Pei-Li Bu

**Affiliations:** National Key Laboratory for Innovation and Transformation of Luobing Theory, The Key Laboratory of Cardiovascular Remodeling and Function Research, Chinese Ministry of Education, Chinese National Health Commission and Chinese Academy of Medical Sciences, Department of Cardiology, Qilu Hospital of Shandong University, Jinan, China; Army Medical University, CHINA

## Abstract

Cardiac remodeling is the primary pathological feature of chronic heart failure (HF). Exploring the characteristics of cardiac remodeling in the very early stages of HF and identifying targets for intervention are essential for discovering novel mechanisms and therapeutic strategies. *Silent mating type information regulation 2 homolog 3 (SIRT3)*, as a major mitochondrial nicotinamide adenine dinucleotide (NAD)-dependent deacetylase, is required for mitochondrial metabolism. However, whether SIRT3 plays a role in cardiac remodeling by regulating the biosynthesis of mitochondrial cardiolipin (CL) is unknown. In this study, we induced pressure overload in wild-type (WT) and SIRT3 knockout (SIRT3^−/−^) mice via transverse aortic constriction (TAC). Compared with WT mouse hearts, the hearts of SIRT3^−/−^ mice exhibited more-pronounced cardiac remodeling and fibrosis, greater reactive oxygen species (ROS) production, decreased mitochondrial-membrane potential (ΔΨm), and abnormal mitochondrial morphology after TAC. Furthermore, SIRT3 deletion aggravated TAC-induced decrease in total CL content, which might be associated with the downregulation of the CL synthesis related enzymes cardiolipin synthase 1 (CRLS1) and phospholipid-lysophospholipid transacylase (TAFAZZIN). In our *in vitro* experiments, SIRT3 overexpression prevented angiotensin II (AngII)- induced aberrant mitochondrial function, CL biosynthesis disorder, and peroxisome proliferator-activated receptor gamma (PPARγ) downregulation in cardiomyocytes; meanwhile, SIRT3 knockdown exacerbated these effects. Moreover, the addition of GW9662, a PPARγ antagonist, partially counteracted the beneficial effects of SIRT3 overexpression. In conclusion, SIRT3 regulated PPARγ-mediated CL biosynthesis, maintained the structure and function of mitochondria, and thereby protected the myocardium against cardiac remodeling.

## 1. Introduction

Cardiac remodeling is the term for adaptive changes in response to mechanical, neurohormonal, and inherited causes of heart failure (HF) that precede the development of the condition [1]. Prevention and treatment of cardiac remodeling can effectively slow the progression of HF and improve patients’ survival rates and quality of life. Under pathological conditions such as pressure overload, a series of changes occur in substrate metabolism, mitochondrial function, energy modulation, and sensing mechanisms of cardiomyocytes to adapt to the pathological conditions and maintain myocardial function. Additional evidence suggests that myocardial metabolic remodeling before HF occurs is closely related to the progression of left-ventricular hypertrophy (LVH) and is important in initiating and maintaining HF [2]. Moreover, energy metabolism disorder can exacerbate cardiac remodeling and accelerate the progression of HF, resulting in increased rates of patient hospitalization and mortality [3]. Cardiac metabolic remodeling has therefore become a novel target and idea for intervention into LVH.

Present research into cardiac metabolic remodeling mainly focuses on changes in the regulation of mitochondrial function. Doenst et al. found that rats with pressure overload induced by thoracic aortic constriction showed diastolic dysfunction after 4 weeks and systolic dysfunction after 6 weeks, but metabolic disorders had already occurred after 2 weeks [4]. Myocardial remodeling is bound up with mitochondrial dysfunction [5]. Another study found that pressure overload could lead to changes in mitochondrial-energy metabolism, which is critical in myocardial remodeling [6]. Investigating the relationship between mitochondrial dysfunction and cardiac remodeling and discovering the substances that link abnormal metabolism with mitochondrial function require deep analysis of the complex structure and role of mitochondria. Recent studies on cardiolipin (CL) have yielded some enlightenment. CL is an essential phospholipid that constitutes the inner membranes of mitochondria and sarcoplasmic reticulum [7]; it maintains the structural integrity of mitochondria, the foundation for oxidative phosphorylation (OXPHOS) and adenosine triphosphate (ATP) production. Reduced and aberrant levels of CL cause structural changes and dysfunction of cardiomyocytic mitochondria [8]. Abnormal CL biosynthesis and remodeling is a common pathological phenomenon in most cardiovascular diseases (CVDs), and its molecular mechanism has received extensive attention. Barth syndrome has been found to feature intracellular CL remodeling disorder accompanied by an increase in monolysocardiolipin (MLCL), an intermediate of CL remodeling, and a decrease in CL [9]. Abnormal CL biosynthesis leads to the destruction of mitochondrial cristae and mitochondrial dysfunction. Studies have shown that the decrease in CL caused by the deletion of the key enzymes involved in CL biosynthesis can lead to cardiomyocytic hypertrophy [10]. These results suggest that changes in CL content and CL remodeling might be important in the pathogenesis of cardiac remodeling. In view of CL’s important physiological functions, can we effectively prevent myocardial mitochondrial dysfunction by regulating the content and biosynthesis of mitochondrial CL? Unfortunately, we cannot yet answer this question because the precise regulatory mechanism of mitochondrial CL is still unknown.

Silent mating type information regulation 2 homolog 3 (sirtuin 3, or SIRT3), a sirtuin family member mainly located in mitochondria, has been reported to have protective effects in various HF phenotypes [11, 12]. In our previous studies, we found that mitochondrial lipid metabolism disorders occurred in the hypertrophic myocardium of SIRT3 knockout (SIRT3^−/−^) mice. SIRT3 overexpression in cardiomyocytes can deacetylate long-chain acyl-coenzyme A (CoA) dehydrogenase (ACAD) and increase its activity, thereby reducing lipid accumulation in hypertrophic cardiomyocytes [13]. These findings indicate that SIRT3 is closely related to lipid metabolism disorders. Screening its downstream lipid-regulatory molecules could, therefore, reveal a key node in the regulation of myocardial lipid remodeling. Recent studies have shown that mitochondrial SIRT3 can regulate CL levels in mouse skeletal muscle; upregulate phospholipid-lysophospholipid transacylase (TAFAZZIN), a CL-remodeling catalytic enzyme; and repress the activities of mitochondrial respiratory-chain (MRC) complexes I and IV with no significant change in total mitochondrial respiratory efficiency [14]. In addition, mitochondrial phospholipid and fatty acid composition changes as phosphatidylcholine (PC)/phosphatidylethanolamine (PE) ratio and arachidonic acid content increases. These results suggest that SIRT3 might play an important role in the synthesis and remodeling of CL. However, the regulatory effect of SIRT3 on CL has not been explored in the myocardial remodeling model induced by pressure overload.

Based on the above-described analysis and previous work, it is unclear whether SIRT3 plays an important role in pressure overload-induced cardiac remodeling by regulating CL biosynthesis and mitochondrial function. Therefore, in this study, we comprehensively analyzed the role of SIRT3 in myocardial remodeling at animal and cellular levels, aiming to explore its role in regulating CL biosynthesis and possible underlying mechanism, as well as its relationship with mitochondrial dysfunction.

## 2. Materials and methods

### 2.1 Animal model

All animal studies were approved by the appropriate ethics committee and performed in accordance with the ethical standards specified in the 1964 Declaration of Helsinki and its later amendments. In addition, all experiments were approved by the ethics boards of Qilu Hospital of Shandong University (Ji’nan, China). We obtained SIRT3^−/−^ (No. 129-SIRT3tm1.1Fwa/J) mice from Jackson Laboratory (Bar Harbor, ME, USA) and wild-type (WT; No. 129S1/SvImJ) mice from Vital River Laboratory Animal Technology Co., Ltd. (Beijing, China) as controls. All mice were male and aged 8 weeks. A cardiac-hypertrophy model was established via transverse aortic constriction (TAC) surgery, which was performed as previously described [13]. We randomly divided SIRT3^−/−^ and WT mice into the following groups (n = 5 each): (1) WT + sham; (2) SIRT3^−/−^ + sham; (3) WT + TAC; and (4) SIRT3^−/−^ + TAC. All animals were euthanized by sodium pentobarbital (0.8%) 4 weeks after surgery, and their hearts were harvested for subsequent analyses.

### 2.2 Echocardiography

We performed echocardiography using a Visual Sonics Vevo 770 imaging system (FUJIFILM VisualSonics, Inc., Toronto, ON, Canada) with a 30-MHz high-frequency transducer. All mice were anesthetized with 2.0% isoflurane (RWD Life Science Co., Ltd., Shenzhen, China). We captured images via motion mode (M-mode), pulse wave Doppler, and tissue Doppler imaging.

### 2.3 Histopathology and immunostaining

The heart tissues were fixed with 4% paraformaldehyde in phosphate buffered saline at room temperature for 48 hours and embedded in paraffin. The samples were cut into 5μm, and stained with hematoxylin/eosin (HE) to evaluate the cardiac histological morphology. Masson’s trichrome staining and Sirius red staining were used to measure cardiac collagen deposition. The slides were incubated overnight at 4°C with the specific primary antibodies against Collagen I (14696-1-AP, Proteintech), Collagen III (AF5457, Affinity), TAFAZZIN (sc365810, Santa Cruz Biotechnology), CRLS1(14845-1-AP, Proteintech) and OPA1(ab157457, Abcam) for immunohistochemistry.

### 2.4 Transmission electron microscopy

Heart samples from the ventricular anterior wall were obtained and fixed in 2.5% glutaraldehyde (pH = 7.2) and 1% osmium tetroxide, and processed as described previously [15]. The slices were viewed with the transmission electron microscope (JEM-1230, JEOL Ltd., Tokyo, Japan).

### 2.5 Cardiolipin content analysis

Cardiolipin content analyses were assessed by enhanced multidimensional mass spectrometry conducted at LipidALL Technologies using a Shimadzu Nexera 20-AD/ ExionLC-AD coupled with Sciex QTRAP 6500 PLUS as reported [16]. Separation of individual lipid classes of polar lipids by normal phase (NP)-HPLC was carried out using a TUP-HB silica column (i.d. 150x2.1 mm, 3 μm) with the following conditions: mobile phase A (chloroform: methanol: ammonium hydroxide, 89.5:10:0.5) and mobile phase B (chloroform: methanol: ammonium hydroxide: water, 55:39:0.5:5.5). Cardiolipin content in cardiomyocytes was assessed by immunofluorescence staining (10N‐nonyl acridine‐orange, A1372; Invitrogen) [17].

### 2.6 Neonatal-rat cardiomyocyte isolation and culture

Neonatal-rat cardiomyocytes (NRCMs) were isolated from neonatal rats as described previously [18]. NRCMs were cultured in Dulbecco’s Modified Eagle Medium (DMEM) supplemented with 10% fetal bovine serum (FBS) and 1% penicillin/streptomycin in an atmosphere of 37°C with 5% CO2.

### 2.7 Plasmids and RNA interference

The entire rat SIRT3 coding region was cloned in pcDNA3.1-3xflag plasmids. Lipofectamine 3000 reagents (Invitrogen, USA) were used for transient transfection of plasmids or siRNA duplexes into NRCMs. Small interfering RNA (siRNA) against SIRT3 and negative siRNA were synthesized by GenePharma (Shanghai, China). The sequence of siRNAs with higher silencing efficiency are as follows: Rat SIRT3 sequence 2: sense 5’-AUGCTCTUUAGGCATACTUG-3’, antisense 5’-GCTUUACTUGATCCGTUACG-3’; Rat SIRT3 sequence 3: sense 5’-GCGUUGUGAAACCUGACAUTT-3’, antisense 5’-AUGUCAGGUUUCAACGCTT-3’. The results about overexpression and interference efficiency of SIRT3 in mRNA and protein levels were shown in S2 and S3 Figs in S1 File.

### 2.8 Determination of mitochondria using Mito-Tracker Red CMXRos Probe

To observe the morphological structure of NRCM mitochondria, we labeled them using Mito-Tracker Red CMXRos (Thermo Fisher) with cardiac troponin I (cTnI; No. 21652-1-AP, Proteintech) and then observed them under a Nikon ELIPSE TE2000-S fluorescence microscope (Nikon, Tokyo, Japan).

### 2.9 JC-1 staining

Tetraethylbenzimidazolylcarbocyanine iodide (JC-1), a lipophilic cationic dye that selectively enters mitochondria, was used to monitor ΔΨm. After removing the culture medium, we rinsed cells twice with phosphate-buffered saline (PBS) and loaded them with 1 ml fresh medium and 1 ml JC-1 stain for 20 min at 37°C with 5% CO2, after which the supernatant was removed. Cells were then washed twice with JC-1 stain (1×), and 2 ml fresh DMEM was added. We then observed and photographed the cells under the Nikon fluorescence microscope.

### 2.10 Western blot analysis

Proteins were harvested from freshly dissected mouse hearts or NRCMs using cell lysis buffer. We incubated membranes overnight with primary antibodies against Collagen I, Collagen III, glyceraldehyde 3-phosphate dehydrogenase (GAPDH; No. TA-08; ZSGB-BIO Technology Co. Ltd), TAFAZZIN (No. A12722, Abclonal Technology), SIRT3 (No. D22A3, Cell Signaling Technology), CRLS1 (No. 14845-1-AP, Proteintech), OPA1 (No. 27733-1-AP; Proteintech), DRP1 (No. sc-271583, Santa Cruz Biotechnology), PPARγ (No. 16643-1-AP; Proteintech), and β-tubulin (No. 66240-1-Ig, Proteintech) overnight at 4°C. Protein bands were visualized via enhanced chemiluminescence (Millipore, Burlington, MA, USA); protein levels were detected using an Amersham Imager 600chemiluminescence reader (Amersham, Chicago, IL, USA). We quantified relative protein levels using ImageJ software (National Institutes of Health, Bethesda, MD, USA).

### 2.11 RNA isolation and real-time quantitative polymerase chain reaction

We extracted total RNA from fresh heart tissues using TRIzol reagent (Invitrogen) per the manufacturer’s protocols. cDNA was produced from 1 mg total RNA per sample using Prime Script RT Reagent Kit with Genomic Deoxyribonucleic Acid (gDNA) Eraser (No. RR047A, TaKaRa Bio, Shiga, Japan) per the manufacturer’s instructions. Polymerase chain reaction (PCR) primer sequences were as follows: *Nppa*, forward 5′-AAACTGAGGGCTCTGCTCG-3′, reverse 5′-CCTGTCAATCCTACCCCCGA-3′; *Nppb*, forward 5′- GAGTCCTTCGGTCTCAAGGC-3′, reverse 5′- TACAGCCCAAACGACTGACG-3′; *Gapdh*, forward 5′-AGGTCGGTGTGAACGGATTTG-3′, reverse 5′-TGTAGACCATGTAGTTGAGGTCA-3′; *Sirt3*, forward 5′-GAGGTTCTTGCTGCATGTGGTTG-3′, reverse 5′-AGTTTCCCGCTGCACAAGGTC-3’.

mRNA levels of genes were analyzed using TB Green Premix Ex Taq (No. RR420A; TaKaRa) per the manufacturer’s instructions.

### 2.12 Measurement of glycolytic/mitochondrial ATP levels

Total and glycolytic ATP were detected using a glycolysis/OXPHOS assay kit (G270, Dojindo, Japan) with or without oligomycin (2.5μm) to assess OXPHOS levels as described previously. Mitochondrial ATP was calculated by subtracting glycolytic ATP from total ATP. Luminenscence was measured using a microplate reader Varioscan Lux (Thermo Fisher Scientific) [19].

### 2.13 Data and statistical analysis

All analyses were performed using GraphPad Prism version 8.0.2 (GraphPad Software, Inc., San Diego, CA, USA). We assessed the normality assumption of the data distribution using the Shapiro-Wilk test. For normally distributed data, an unpaired, two-tailed Student’s t test was performed to determine the statistical difference between two groups, and one- or two-way analysis of variance (ANOVA) was performed to determine the statistical difference between multiple groups. For data with non-normal distribution, we performed the Kruskal-Wallis test for nonparametric statistical analysis. Statistical significance was set to P < 0.05. All data are represented as mean ± standard error of the mean (SEM).

## 3. Results

### 3.1 SIRT3 deletion aggravated pathological development of cardiac remodeling induced by TAC

**Fig 1:**
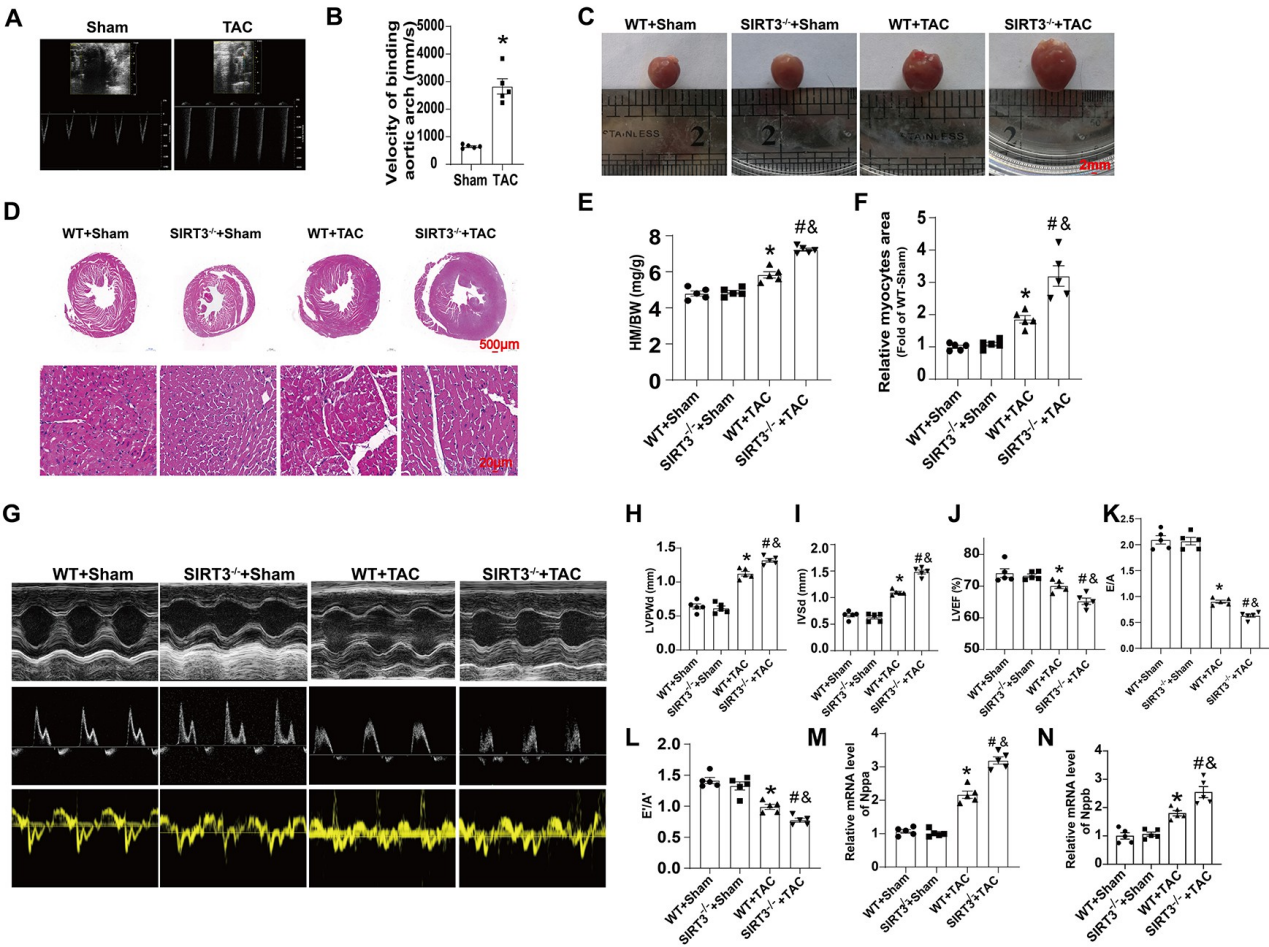
SIRT3 deletion aggravated pathological development of cardiac remodeling induced by TAC. (A) Velocity of binding aortic arch in Sham- or TAC-treated mice. (B) Statistical analysis of velocity of binding aortic arch of Sham- or TAC-treated mice (n = 5). (C) Representative gross appearance of Sham- or TAC-treated WT and SIRT3^-/-^ mice heart. (D) HE-stained cross-sections of mouse hearts. (E) Heart mass (HM) normalized to body weight (BW). (F) Relative myocyte size by HE staining. (G) Representative echocardiographic images of M-mode, pulse-wave Doppler and tissue Doppler in 4 groups of mice. (H-L). Statistical analysis of LVPWd, IVSd, LVEF, E/A and E’/A’. M-N. Statistical analysis of relative mRNA level of Nppa and Nppb. n = 5 per group. *p<0.05 *vs*. WT+Sham group, &p<0.05 *vs*. SIRT3^-/-^+Sham group, #p<0.05 *vs*. WT+TAC group.

We performed TAC surgery and sham operations on both WT and SIRT3^−/−^ mice. Twenty-eight days after surgery, modeling success was demonstrated by an increased aortic-peak flow velocity (Fig 1A and 1B) measured at the site of constriction by Doppler UCG. Mouse heart mass-to-body weight (HM/BW) ratios also significantly increased after TAC (Fig 1C and 1E), more notably in the SIRT3^−/−^ group. Compared with sham operation mice, left-ventricular posterior-wall end-diastole (LVPWd) and interventricular-septum end-diastole (IVSd) thickness were significantly increased after TAC surgery, with a significant decrease in E/A and E′/A′ indicating diastolic dysfunction (Fig 1G–1L). TAC-induced cardiac diastolic dysfunction was further exacerbated in SIRT3^−/−^ mice compared with WT mice. Hematoxylin and eosin (H&E) staining showed that TAC increased the size of cardiomyocytes, which was further promoted by SIRT3 deficiency (Fig 1D and 1F). We then performed mRNA characterization of hypertrophy-related markers to confirm our findings at the molecular level. SIRT3^−/−^ hearts demonstrated higher mRNA expression of Nppa and Nppb than WT hearts after TAC (Fig 1M and 1N). These data suggested that SIRT3 deletion aggravated TAC-induced cardiac remodeling.

### 3.2 SIRT3 deletion aggravated TAC-induced myocardial fibrosis

**Fig 2:**
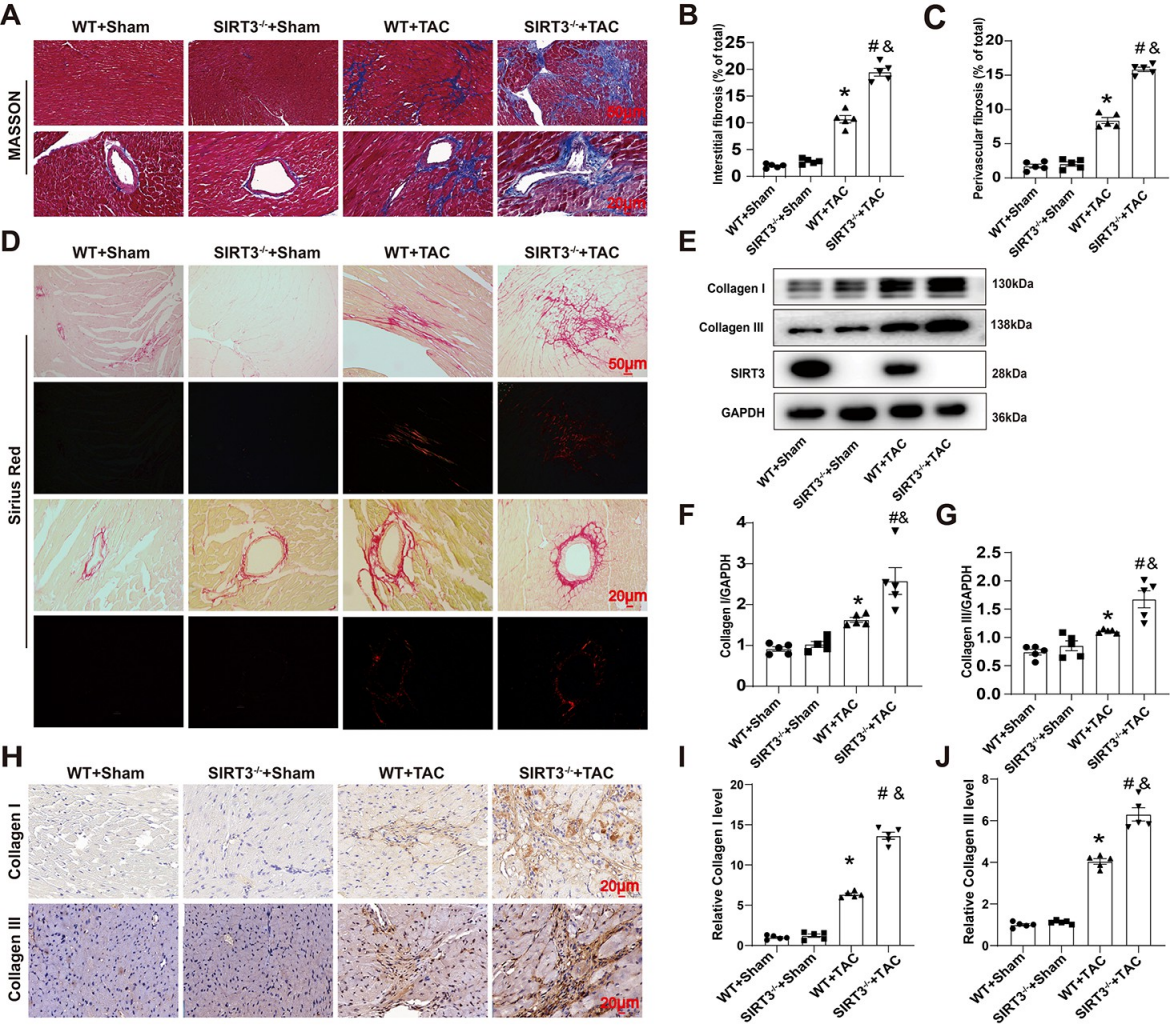
SIRT3 deletion aggravated TAC-induced myocardial fibrosis. (A) Masson-stained heart sections. (B) Statistical analysis of interstitial fibrosis fraction. (C) Statistical analysis of perivascular fibrosis fraction. (D) Sirius red‐stained heart sections. (E) Representative Western blot images of Collagen I, Collagen III and SIRT3 protein expressions in the myocardium. (F-G). Quantifications of Collagen I, Collagen III protein expressions in the myocardium. (H-J) Representative immunohistochemical staining and quantifications of Collagen I, Collagen III and TUNEL in the myocardium. n = 5 per group. *p<0.05 *vs*. WT+Sham group, &p<0.05 *vs*. SIRT3^-/-^+Sham group, #p<0.05 *vs*. WT+TAC group.

In addition to cardiac diastolic dysfunction, cardiomyocytic hypertrophy, and activation of the fetal gene program, interstitial fibrosis is also involved in myocardial remodeling [20]. Therefore, we evaluated the effect of SIRT3 on TAC-induced cardiac fibrosis. Masson and Sirius Red staining showed obvious interstitial and peritubular fibrosis in WT mice after TAC, as manifested by increased interstitial and peritubular collagen deposition; this fibrosis was further aggravated after SIRT3 deletion (Fig 2A–2D). Western blot analysis of myocardium from all groups of mice revealed that upregulation of Collagen I and Collagen III protein levels induced by TAC surgery was further promoted after SIRT3 deletion (Fig 2E–2G). Similar results were also obtained from immunohistochemical (IHC) analysis of Collagen I and Collagen III deposition (Fig 2H–2J). Taken together, these results indicated that SIRT3 deletion aggravated TAC-induced cardiac fibrosis.

### 3.3 SIRT3 deletion impaired mitochondrial structure and cardiolipin biosynthesis

**Fig 3:**
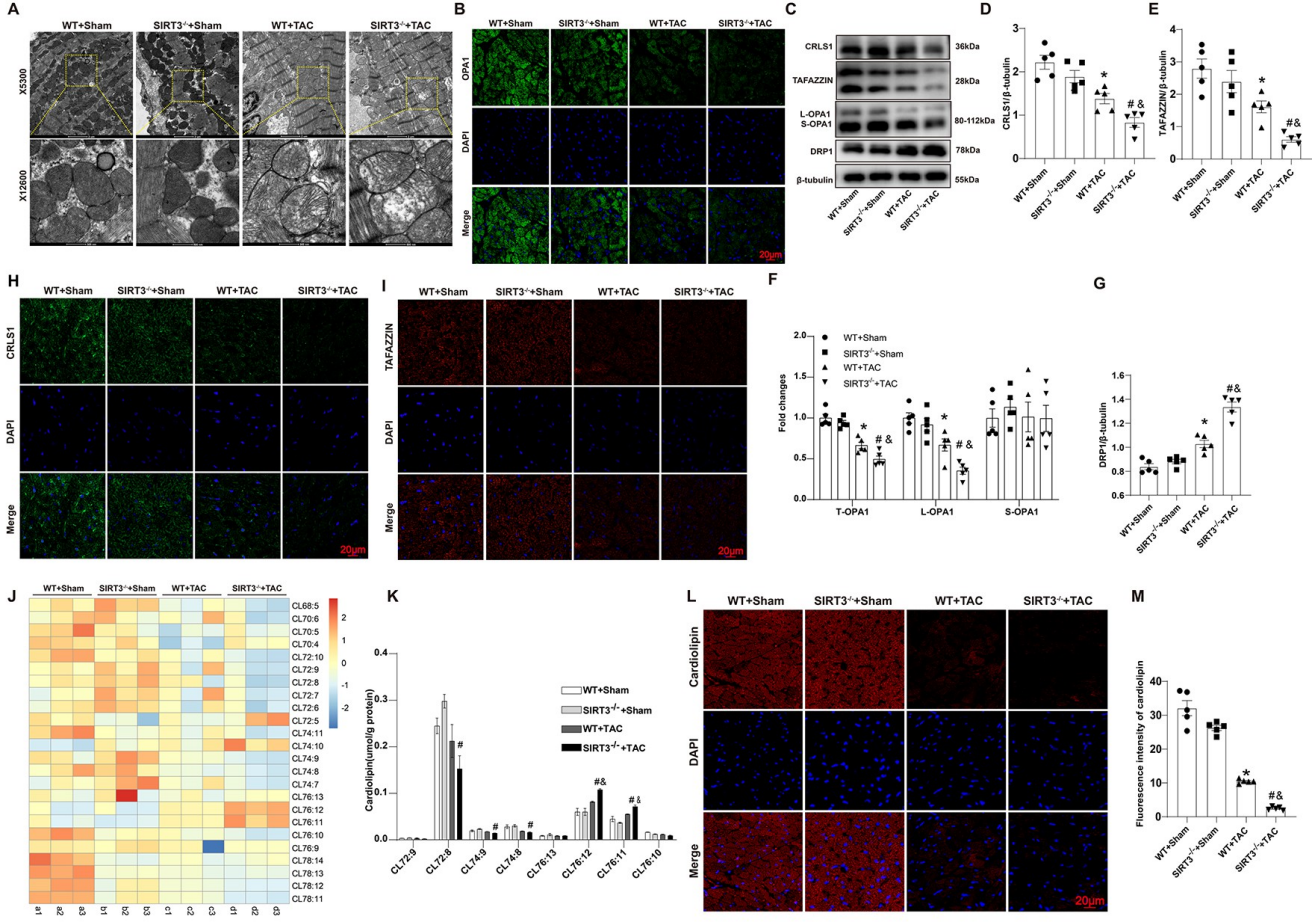
SIRT3 deletion impaired mitochondrial structure and cardiolipin biosynthesis. (A) Representative transmission electron microscope images. (B) Representative images of immunofluorescent staining of OPA1 in the myocardium. (C-G) Representative Western blot images and quantifications of CRLS1, TAFAZZIN, OPA1(L-OPA1 and S-OPA1) and DRP1 protein expressions in the myocardium. (H-I) Representative immunofluorescentimmunohistochemical staining of CRLS1 and TAFAZZIN expression in the myocardium. (J) Heatmap of cardiolipin content in four groups (n = 3 per group). (K) Statistical analysis of content of different cardiolipin species (n = 3 per group). (L-M) Representative immunofluorescent staining and quantifications of cardiolipin expression in the myocardium. n = 5 per group. *p<0.05 *vs*. WT+Sham group, &p<0.05 *vs*. SIRT3^-/-^+Sham group, #p<0.05 *vs*. WT+TAC group.

Based on the crucial role played by mitochondrial homeostasis of cardiomyocytic metabolism in cardiac metabolic remodeling, we further examined changes caused by SIRT3 deletion in the mitochondrial structure and function of mouse hearts. Under the TEM, we observed that after TAC, mitochondria in WT mice were significantly swelling, with a disordered arrangement and reduced cristae; these findings were much more obvious in SIRT3^−/−^ mice after TAC (Fig 3A). OPA1, which anchors to the inner mitochondrial membrane (IMM), is an obligatory protein for mitochondrial fusion. Therefore, we detected OPA1 levels in both groups. We found that total OPA1(T-OPA1) was reduced after TAC, and such downregulation was more noticeable in the SIRT3^−/−^ than in the WT group (Fig 3B, 3C and 3F). Notably, OPA1 can be extensively cleaved that causes long isoform OPA1(L-OPA1) loss and OPA1 dysfunction under pathologic stress. Evidence have shown that L-OPA1 is a critical role in driving inner membrane fusion [21]. In this study, a significant downregulation of L-OPA1 was also observed, which was more obviously in the SIRT3^-/-^ group. However, the change of short isoform OPA1 (S-OPA1) was less obvious among all groups. (Fig 3C and 3F). Besides, we also focused on progress of mitochondrial fission. DRP1, a marker for mitochondrial fission, was tested. The results showed that DRP1 was upregulated after TAC and was further promoted after SIRT3 deletion (Fig 3C and 3G). The normal structure and function of mitochondria depend on the normal production and remodeling of CL; therefore, we also assessed changes in CL biosynthesis in mouse myocardial tissues. CL content analysis showed that in addition to a decrease in overall CL abundance, CL species also obviously changed: levels of CL72:8, CL74:8, and CL74:9 decreased substantially after TAC (Fig 3J and 3K). Immunofluorescence (IF) staining revealed that total CL decreased markedly after TAC, and the decrease was more pronounced in the SIRT3^−/−^ than in the WT group (Fig 3L and 3M). CL biosynthesis involves a complex process regulated by many enzymes. We detected the expression of CRLS1 and TAFAZZIN, enzymes that are key to CL biosynthesis and remodeling. Western blot showed that protein levels of both enzymes were obviously decreased in both mouse groups after TAC, more so in the SIRT3^−/−^ group (Fig 3C–3E). These findings were consistent with IF imaging results (Fig 3H and 3I). Taken together, these results indicated that SIRT3 deletion might impair mitochondrial homeostasis by blocking CL biosynthesis in mouse hearts after TAC.

### 3.4 SIRT3 affected mitochondrial function in AngII-induced cardiomyocytes

**Fig 4:**
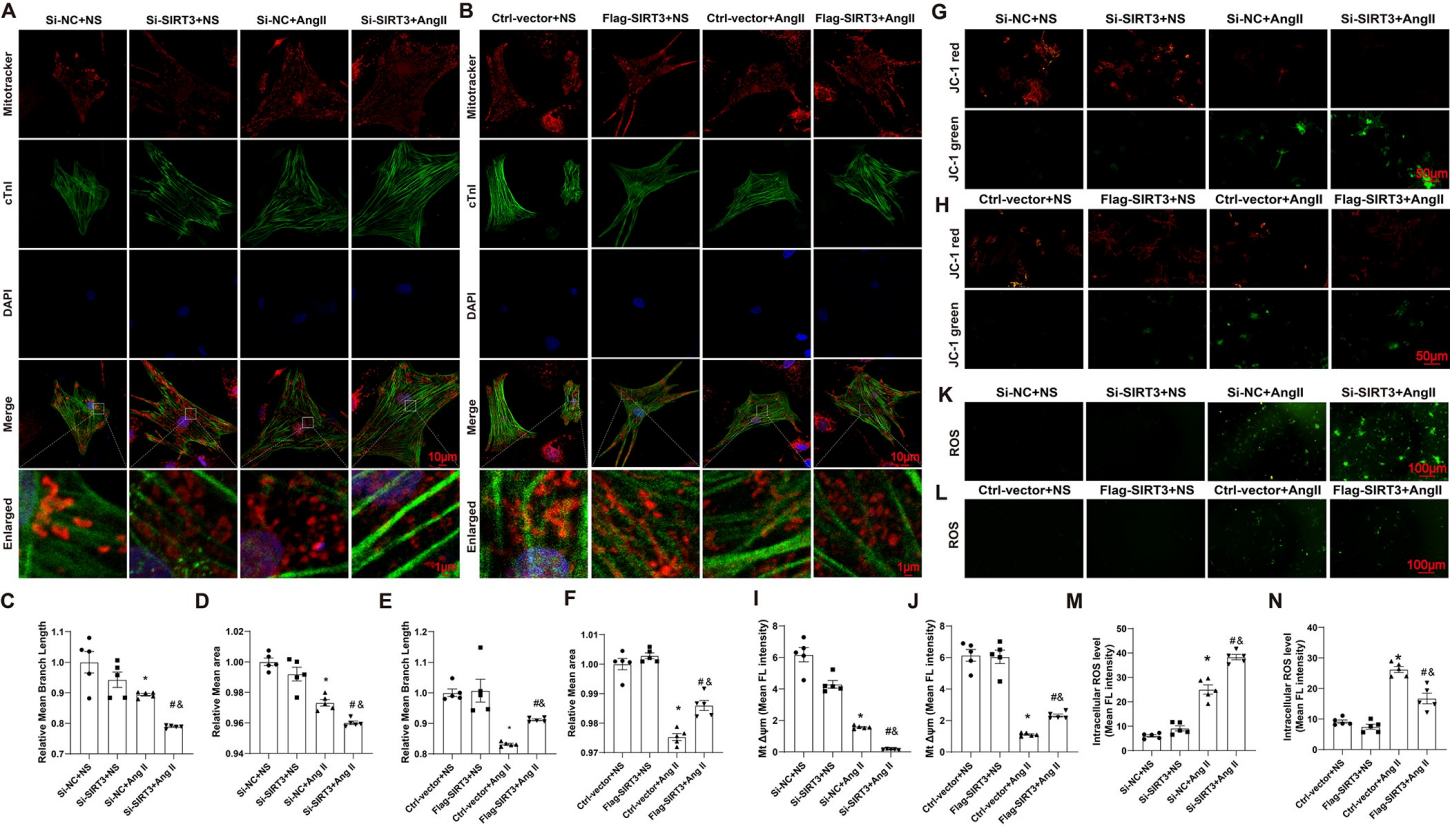
SIRT3 affected mitochondrial function in AngII-induced cardiomyocytes. (A-B) Representative images of mitochondrial morphology in NRCMs. (C-F) Quantification of relative mean branch length and relative mean area of mitochondrion in NRCMs. (G-J) Representative images and quantification of Δψm in NRCMs. Δψm was stained with JC-1 followed by fluroscence analysis. (K-N) Representative images and quantification of intracellular ROS in NRCMs. n = 5 per group, *p<0.05 *vs*. Si-NC+NS or Ctrl-vector+NS group, &p<0.05 *vs*. Si-SIRT3+NS or Flag-SIRT3+NS group, #p<0.05 vs. Si-NC+AngII group or Ctrl-vector +AngII group.

To explore the effect of SIRT3 on mitochondrial function, we isolated NRCMs and treated them with AngII to mimic the pathological process of cardiomyocytic hypertrophy. More mitochondrial fragments and globular mitochondria (shorter branches, smaller area) formed in AngII-treated NRCMs than in NS-treated NRCMs, accompanied by aberrant mitochondrial function demonstrated by decreased Δψm and increased reactive oxygen species (ROS) levels. The AngII-induced increase in abnormal mitochondrial patterns was more obvious in the Si-SIRT3 group (Fig 4A, 4C and 4D). In contrast, SIRT3 overexpression alleviated AngII-induced mitochondrial-membrane rupture, leading mitochondria to be more filamentous and branched (Fig 4B, 4E and 4F). Moreover, knockdown of SIRT3 facilitated the AngII-induced decrease in Δψm and increase in ROS, whereas overexpression of SIRT3 had the opposite effect (Fig 4G–4N). The decrease in OXPHOS-derived ATP induced by AngII was aggravated in Si-SIRT3 group, which was prevented by overexpression of SIRT3 (S1 Fig in S1 File). It was also observed that glycolysis of cardiomyocytes increased shown by lactate production was higher in AngII treatment group, which was exacerbated by SIRT3 knockdown and repressed by SIRT3 overexpression. These results indicated that SIRT3 played a protective role in AngII-induced mitochondrial dysfunction in cardiomyocytes.

### 3.5 SIRT3 affected CL content and CL synthesis related enzymes in AngII-treated cardiomyocytes

**Fig 5:**
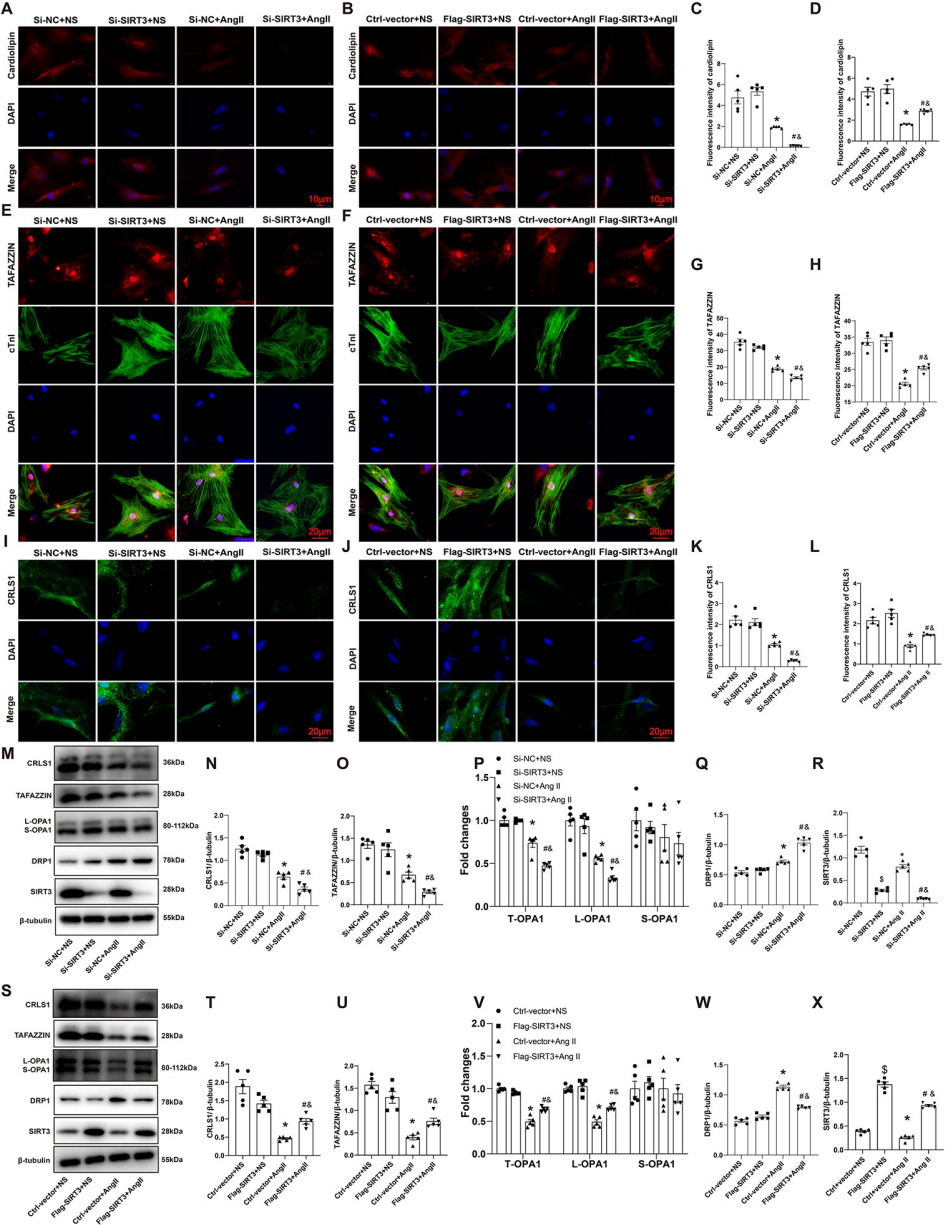
SIRT3 affected CL content and CL synthesis related enzymes in AngII-treated cardiomyocytes. (A-D) Representative immunofluorescence staining images and quantifications of cardiolipin in NRCMs. (E-H) Representative immunofluorescence staining images and quantifications of TAFAZZIN in NRCMs. (I-L) Representative immunofluorescence staining images and quantifications of CRLS1 in NRCMs. (M-X) Representative western blot images and quantifications of CRLS1, TAFAZZIN, OPA1(L-OPA1 and S-OPA1), DRP1 and SIRT3 protein expressions in NRCMs. n = 5 per group, *p<0.05 *vs*. Si-NC+NS or Ctrl-vector+NS group, &p<0.05 *vs*. Si-SIRT3+NS or Flag-SIRT3+NS group, #p<0.05 vs. Si-NC+AngII group or Ctrl-vector +AngII group.

Next, we explored the regulatory role that SIRT3 played in CL biosynthesis in NRCMs. IF staining showed that total CL level was downregulated in AngII-treated NRCMs. SIRT3 downregulation further exacerbated this decrease, while overexpression of SIRT3 significantly repressed the AngII-induced total reduction of CL (Fig 5A–5D). IF staining (Fig 5E–5L) and western blot (Fig 5M–5O and 5S–5U) results showed that AngII treatment downregulated CRLS1 and TAFAZZIN levels in NRCMs, which was promoted by SIRT3 downregulation and prevented by SIRT3 overexpression. Levels of OPA1 and L-OPA1 were obviously reduced under AngII stimulation. SIRT3 downregulation further exacerbated this reduction, which was prevented by overexpression of SIRT3 (Fig 5M, 5P, 5S and 5U). Besides, DRP1 was upregulated by AngII treatment, which was promoted by SIRT3 knockdown and prevented by SIRT3 overexpression (Fig 5M, 5Q, 5S and 5W). These results suggested that SIRT3 regulated biosynthesis of mitochondrial CL in AngII-treated NRCMs, a mechanism that might underlie SIRT3’s regulation of mitochondrial function.

### 3.6 SIRT3 regulated CL biosynthesis by modulating PPARγ activation

**Fig 6:**
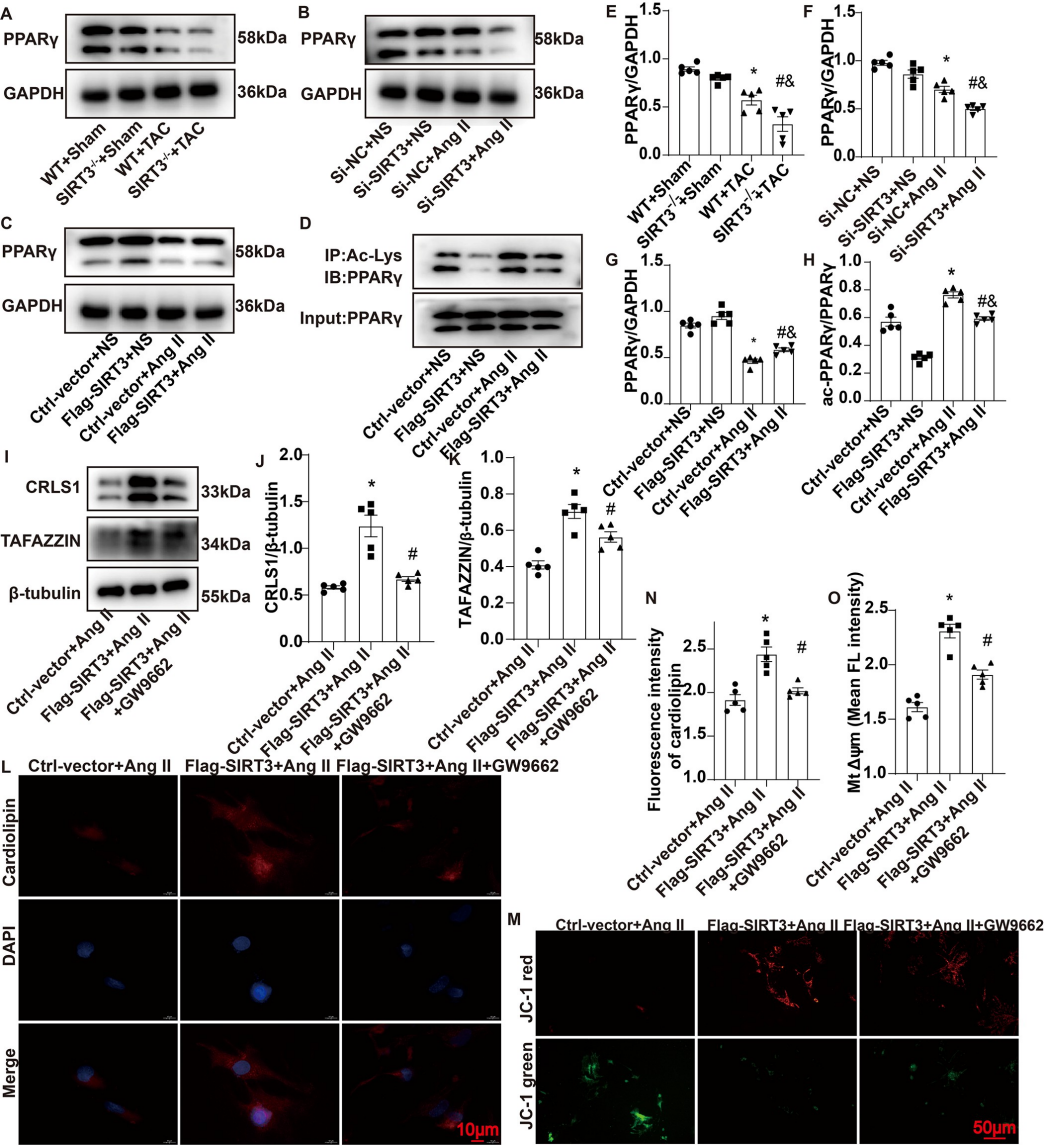
SIRT3 regulated CL biosynthesis by modulating PPARγ activation. (A and E) Representative Western blot images and quantifications of PPARγ protein expressions in the mouse myocardium. (B, C, F and G). Representative Western blot images and quantifications of PPARγ protein expressions in NRCMs. (D and H). Representative Western blot images and quantifications of acetylated PPARγ expressions in NRCMs. (I-K) Representative Western blot images and quantifications of CRLS1 and TAFAZZIN protein expressions in NRCMs. (L and N) Representative immunofluorescence staining images and quantifications of cardiolipin in NRCMs. (M and O) Representative images and quantification of Δψm in NRCMs. Δψm was stained with JC-1 followed by fluroscence analysis. n = 5 per group, *p<0.05 *vs*. WT+ Sham group (E), Si-NC+NS group (F), Ctrl-vector+NS (G, H), Ctrl-vector+ AngII (J, K, N, O); &p<0.05 *vs*. SIRT3^-/-^+Sham group (E), Si-SIRT3+NS group(F), Flag-SIRT3+NS (G, H); #p<0.05 *vs*. WT+TAC group (E), Si-NC+AngII group (F) or Ctrl-vector+AngII group (G, H), Flag-SIRT3+AngII group (J, K, N, O).

PPARγ, a member of the nuclear hormone receptor PPAR superfamily, is a key regulator of adipogenesis [22]. We speculated that PPARγ might be involved in SIRT3’s regulation of the CL pathway; therefore, our next experiments explored the role of SIRT3 in PPARγ expression and activation. Compared with sham operation mice, PPARγ protein levels decreased in the myocardium from WT mice following TAC, and SIRT3 deletion further promoted this decrease (Fig 6A and 6E). Consistently, SIRT3 siRNA induced PPARγ downregulation in response to AngII treatment in NRCMs, whereas SIRT3 overexpression had the opposite effect (Fig 6B, 6C, 6F and 6G). Given the deacetylase activity of SIRT3, we also examined the acetylation level of PPARγ. Immunoprecipitation results showed that acetylated PPARγ increased in NRCMs after AngII treatment, while SIRT3 overexpression inhibited AngII-induced PPARγ acetylation (Fig 6D and 6H). These results indicated that SIRT3 regulated PPARγ expression and acetylation in response to AngII, which might be a mechanism by which SIRT3 affects CL biosynthesis. Then, we introduced a highly specific PPARγ antagonist, GW9662 [23], to verify the above-described finding. GW9662 partially abrogated the protective effects of SIRT3 overexpression against AngII-induced CL biosynthesis abnormalities and mitochondrial dysfunction in NRCMs, as evidenced by decrease in TAFAZZIN and CRLS1 protein levels, Δψm and total CL (Fig 6I–6O). These data suggested that SIRT3 regulated CL biosynthesis and mitochondrial function at least partly by modulating PPARγ activation.

## 4. Discussion

**Fig 7:**
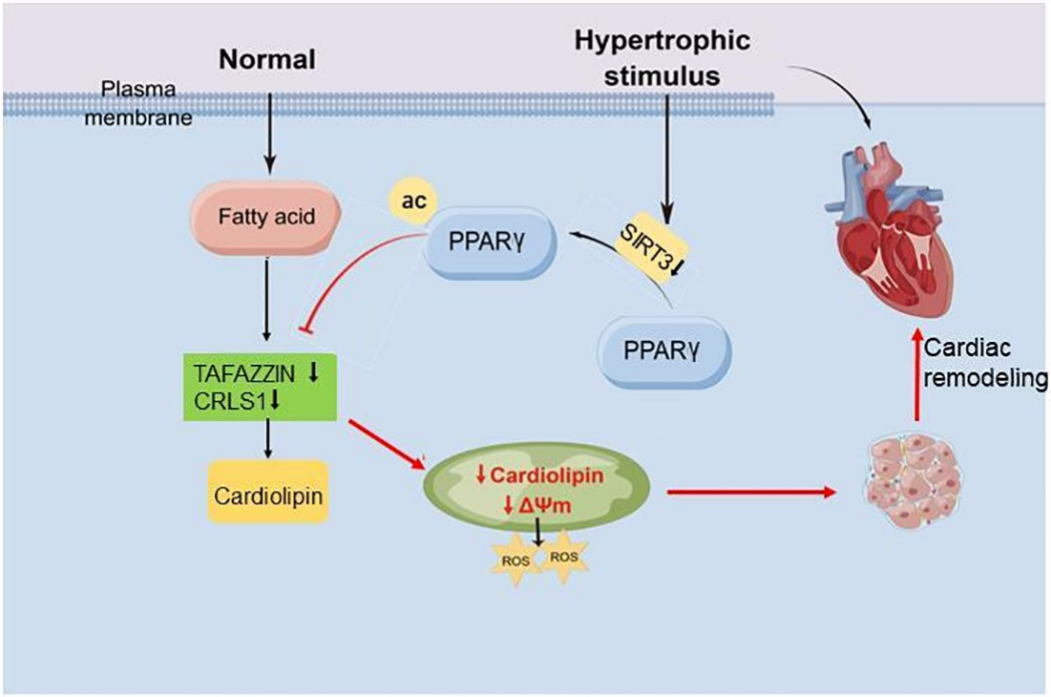
Diagram for the role of SIRT3 in CL biosynthesis regulation and cardiac remodeling.

This study provided evidence that SIRT3 played an important role in cardiac metabolic remodeling by regulating cardiolipin content. Our data showed that SIRT3 was necessary for maintaining normal mitochondrial morphology and function. Hearts of SIRT3^−/−^ mice exhibited more swelling mitochondria and less normal mitochondrial function after TAC, consistent with their higher level of ROS production. These results indicated that SIRT3 was involved in regulating CL under TAC or AngII stimulation, which might be associated with the regulation of the CL biosynthesis-related enzymes CRLS1 and TAFAZZIN. Expression of both these enzymes decreased with SIRT3 siRNA under AngII stimulation and was rescued by SIRT3 overexpression. We then assessed PPARγ’s role in SIRT3’s regulation of CL. The results showed that acetylation of PPARγ increased under AngII stimulation. SIRT3 overexpression reduced PPARγ acetylation and increased CL levels in NRCMs compared with the control group under AngII treatment. Effects of SIRT3 overexpression on CRLS1 and TAFAZZIN expression, as well as on CL levels and mitochondrial function, were partially abrogated by GW9662. These findings showed that SIRT3 regulated CL biosynthesis and mitochondrial function by activating PPARγ (Fig 7).

Cardiac metabolism involves substrate utilization, OXPHOS, and ATP transfer and utilization. Under pathological conditions, cardiac metabolic remodeling can be triggered to maintain ATP balance. In hypertrophic hearts, fatty acid oxidation, glucose utilization, and mitochondrial-energy metabolism decrease while glycolysis increases. In this study, mice after TAC surgery exhibited cardiac diastolic dysfunction and fibrosis, especially in the SIRT3^−/−^ group. We observed abnormal mitochondrial morphology and function in the TAC-treated group, as evidenced by a disordered arrangement and reduced cristae as well as reduced ΔΨm and increased ROS; these changes were much more obvious in the SIRT3^−/−^ group. CL is a crucial component of mitochondria that stabilizes the structure and function of the mitochondrial-electron transport chain and regulates mitochondrial dynamics and crista structure. Mitochondrial morphology and function rely on normal CL content and metabolism [24]. The content and profile of CL change in many diseases. Previous research has demonstrated that CL content decreases and CL profile changes in catecholamine-induced cardiac-damage and HF models [25–27]. In our work, total CL in mouse hearts after TAC was reduced compared with hearts from the sham operation group, and the reduction was more significant in the SIRT3^−/−^ group. CL72:8, the most abundant CL species, modulates mitochondrial morphology and energy production. After TAC, we found CL72:8 levels to be obviously reduced and CL76:11’s level increased, consistent with previous reports on heart diseases. A decrease in CL72:8 is related to cytochrome c oxidase activity reduction and mitochondrial-respiration dysfunction [25]. Our results suggested that impaired CL biosynthesis and remodeling might lead to impaired mitochondrial morphology and function. Taken together, the above-mentioned findings indicated that SIRT3 played a considerable role in CL regulation and that the underlying mechanism is worth further exploration.

OPA1, a dynamin-like GTPase localized in the inner mitochondrial membrane, plays a crucial role in regulating inner membrane fusion and maintaining the structural integrity of mitochondrial cristae. There are various OPA1 isoforms generated by alternative splicing and proteolytic processing. The L-OPA1 can be cleaved into S-OPA1 by the mitochondrial proteases [28]. Evidence have shown that L-OPA1 is a critical role in driving inner membrane fusion [21]. In contrast, accumulation of S-OPA1 will contribute to mitochondrial fission. In our study, the levels of total OPA1 and L-OPA1decreased after TAC, which were more obviously in SIRT3 deletion group. In AngII-treated cardiomyocytes, L-OPA1 was reduced, which aggravated after SIRT3 knockdown, and prevented with SIRT3 overexpression. The dynamin-related protein 1 (DRP1) is the executioner of mitochondrial fission [29]. Excessive activation and accumulation of DRP1 can contribute to pathological fission. In this study, DRP1 was upregulated after TAC, which was further promoted by the SIRT3 deletion. In AngII-treated cardiomyocytes, DRP1 was increased, which was promoted after SIRT3 knockdown, and prevented by SIRT3 overexpression. The results suggested that expression of SIRT3 may involve in regulating the process of mitochondrial fusion and fission by affecting OPA1 and DRP1, thereby regulate mitochondrial function.

CL metabolism in mitochondria is divided into two main steps: biogenesis and remodeling [29]. CRLS1 is involved in CL synthesis by converting phosphatidylglycerol (PG) into CL. CRLS1 mutation results in CL deficiency and changes in mitochondrial morphology [24, 30]. CL remodeling critically depends on TAFAZZIN, a phospholipid-lysophospholipid transacylase that can catalyze nascent CL into its mature form, which contains longer and polyunsaturated acyl chains. Mutation in TAFAZZIN causes inefficient transacylation, resulting in decreased amounts of mature CL [31]. SIRT6, another member of the sirtuin family, has been documented to regulate CL biosynthesis de novo by interacting with p53 [32]. SIRT3 is a major mitochondrial-protein deacetylase. Considering the important role of SIRT3 in lipid metabolism and mitochondrial homeostasis, we speculated that it might be involved in the regulation of CL biosynthesis. Our results revealed that CL content was reduced in the hearts of mice after TAC, especially in SIRT3^−/−^ mice. Moreover, SIRT3 was involved in regulating the expression of CRLS1 and TAFAZZIN. In cardiomyocytes under AngII stimulation, CL content decreased, accompanied by reductions in CRLS1 and TAFAZZIN, which were more obvious after SIRT3 downregulation; meanwhile, the expression of the two enzymes increased after SIRT3 overexpression. Collectively, these results suggested that SIRT3 regulated CL content by regulating enzymes in CL biosynthesis, thereby modulating mitochondrial morphology and function and affecting energy metabolism.

PPARγ is a ligand-activated nuclear transcription factor involved in the control of various aspects of lipid metabolism [33]. Therefore, we next explored whether SIRT3 modulated CL metabolism by regulating PPARγ. Previous studies have reported that PPARγ function is regulated by post-translational modifications, including SUMOylation, phosphorylation, O-GlcNAcylation, and acetylation; according to these researches, different members of the sirtuin family regulate PPARγ by different mechanisms. SIRT1 binds and deacetylates PPARγ to promote the browning of white adipose tissue and energy utilization, while SIRT1 overexpression has no significant effect on PPARγ expression in mice. Deacetylation of PPARγ can activate its transcriptional activity by promoting recruitment of the co-activator PR/SET domain 16 (PRDM16) and the clearance of inhibitory factor [34]. In addition, SIRT6 inhibits PPARγ expression at the transcriptional level and coordinates endothelial fatty acid uptake under pathological conditions to reduce diabetes-associated HF with preserved ejection fraction (HFpEF) [35]. However, very few studies have addressed its role in CL biosynthesis, let alone its association with SIRT3-mediated CL biosynthesis. In our present work, PPARγ acetylation was elevated under AngII stimulation and decreased after SIRT3 overexpression. These results suggested that SIRT3 might exert its effect via PPARγ deacetylation. We used a PPARγ antagonist, GW9662, to demonstrate that PPARγ was required for SIRT3-mediated protection against AngII-induced abnormal CL biosynthesis and mitochondrial dysfunction in cardiomyocytes. GW9662 partially abrogated the improvements in CRLS1 and TAFFAZIN expression as well as CL levels and mitochondrial function via SIRT3 overexpression. This finding was in line with a previous report by Vas et al., who discovered a coactivation relationship between SIRT3 and PPARγ by analyzing the differential expression of adipogenesis-related genes centered on PPARγ [36]. The differential effects of SIRT3, SIRT1, and SIRT6 on PPARγ might be due to different interacting molecules or different subcellular localization, which depend on cellular context.

Despite these novel findings, this study had certain limitations. It is not clear how SIRT3 regulates PPARγ activation, whether by direct activation or through modulation of other signaling pathways, or whether SIRT3 could deacetylate and affect the stability of CL-related metabolic enzymes. These points will require further investigation on our part.

In summary, we found that SIRT3 modulated PPARγ activation, regulated CL metabolism, and maintained the structure and functions of mitochondria, thereby protecting the myocardium against hypertrophic stimuli.

## Supporting information

S1 File Fig S1. SIRT3 affected OXPHOS and glycolysis in NRCMs, Fig S2. Overexpression and interference efficiency of SIRT3 in NRCMs, Fig S3. Overexpression efficiency of SIRT3 in NRCMs.
(DOCX)

S1 Data Raw images. 10.1371/journal.pone.0301990.
(XLSX)

S1 Raw images . (PDF)
