# Supplementary material for: SIRT3 regulates cardiolipin biosynthesis in pressure overload-induced cardiac remodeling by PPARγ-mediated mechanism
Source: PLoS One. 2024 Apr 16;19(4):e0301990. doi: 10.1371/journal.pone.0301990 (PMC11020683; doi:10.1371/journal.pone.0301990)
Supplement: S1 File — Fig S1. SIRT3 affected OXPHOS and glycolysis in NRCMs, Fig S2. Overexpression and interference efficiency of SIRT3 in NRCMs, Fig S3. Overexpression efficiency of SIRT3 in NRCMs. (DOCX) [file pone.0301990.s001.docx]

**ONLINE** **SUPPLEMENTAL MATERIALS**

**SIRT3 regulates cardiolipin biosynthesis in pressure overload-induced cardiac remodeling by a PPARγ-mediated mechanism**

Ling-Xin Liu, Xue-Hui Zheng, Jing-Han Hai, Chun-Mei Zhang, Yun Ti, Tong-Shuai Chen*, Pei-Li Bu*

*National Key Laboratory for Innovation and Transformation of Luobing Theory; The Key Laboratory of Cardiovascular Remodeling and Function Research, Chinese Ministry of Education, Chinese National Health Commission and Chinese Academy of Medical Sciences; Department of Cardiology, Qilu Hospital of Shandong University, Jinan, China*

*Correspondence: Tong-Shuai Chen([chentongshuai@163.com](mailto:chentongshuai@163.com)); Pei-Li Bu ([bupeili@outlook.com](mailto:bupeili@outlook.com))


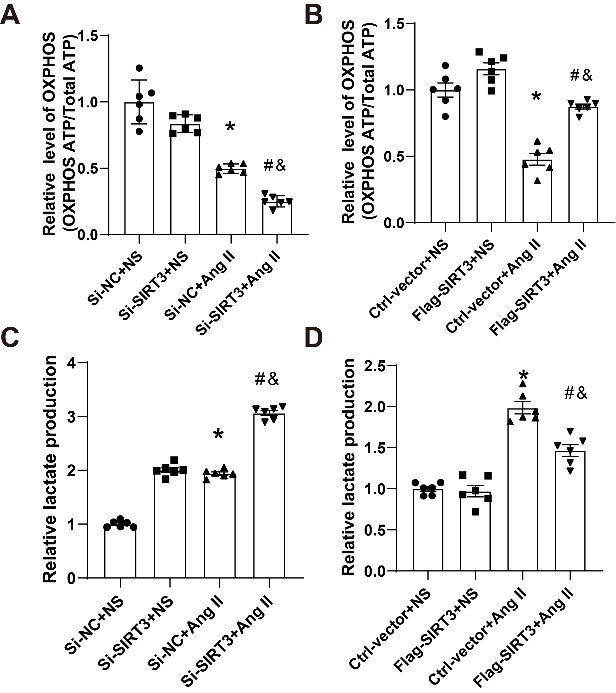


**Figure S1. SIRT3 affected OXPHOS and glycolysis in NRCMs.**

(A-B) Quantification of relative level of OXPHOS (OXPHOS ATP/total ATP) in NRCMs. (C-D) Quantification of relative lactate production in NRCMs. n=6 per group, *p<0.05 vs. Si-NC+NS or Ctrl-vector+NS group, &p<0.05 vs. Si-SIRT3+NS or Flag-SIRT3+NS group, #p<0.05 *vs.* Si-NC+AngⅡ group or Ctrl-vector+AngⅡ group.


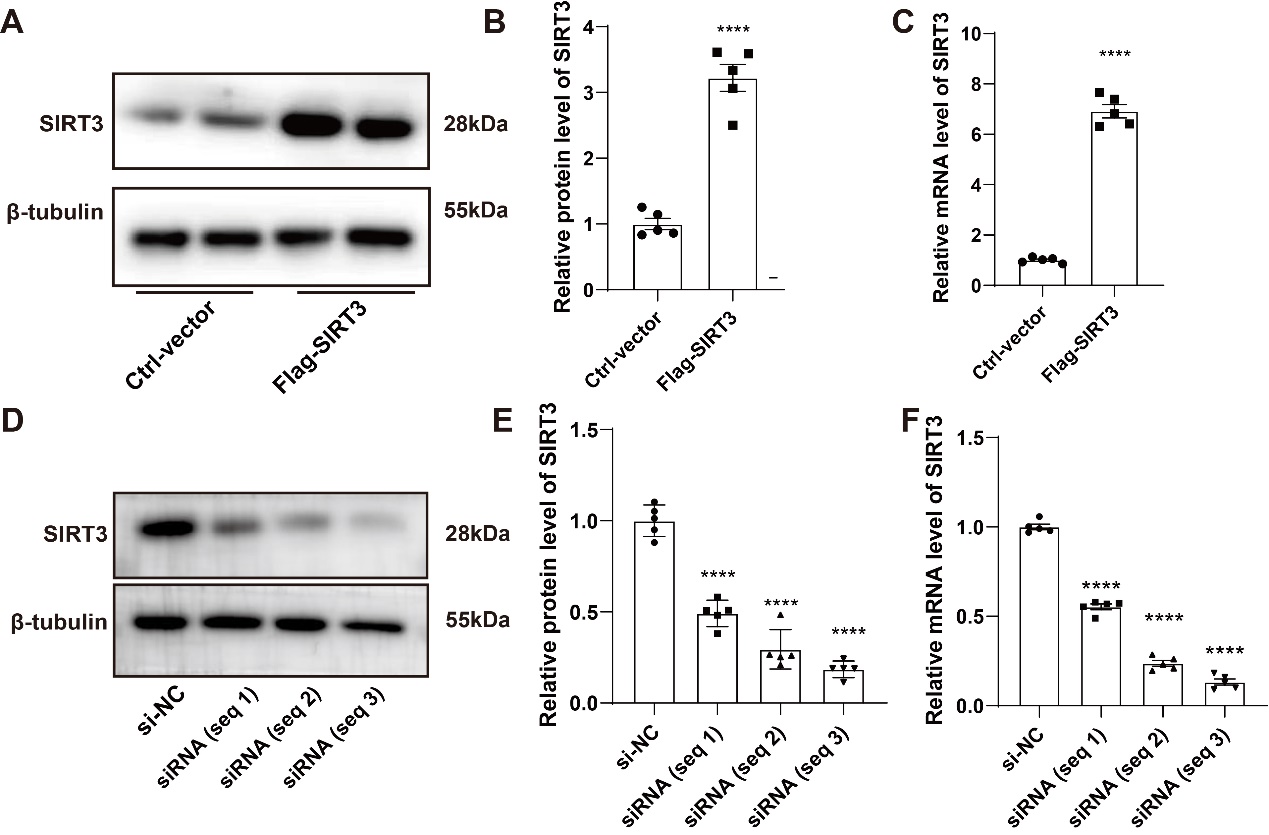


**Figure S2.** **Overexpression and** **interference efficiency of SIRT3 in NRCMs.** (A-B) Representative western blot images and quantification analysis of SIRT3 in plasmid transfection experiment. (C) RT-PCR analysis of SIRT3 mRNA level in plasmid transfection experiment. (D-E) Representative western blot images and quantification analysis of SIRT3 in SiRNA interference experiment. (F) RT-PCR analysis of SIRT3 mRNA level in SiRNA interference experiment. n=5 per group, *****P* < 0.0001 *vs.* Ctrl-vector or Si-NC.
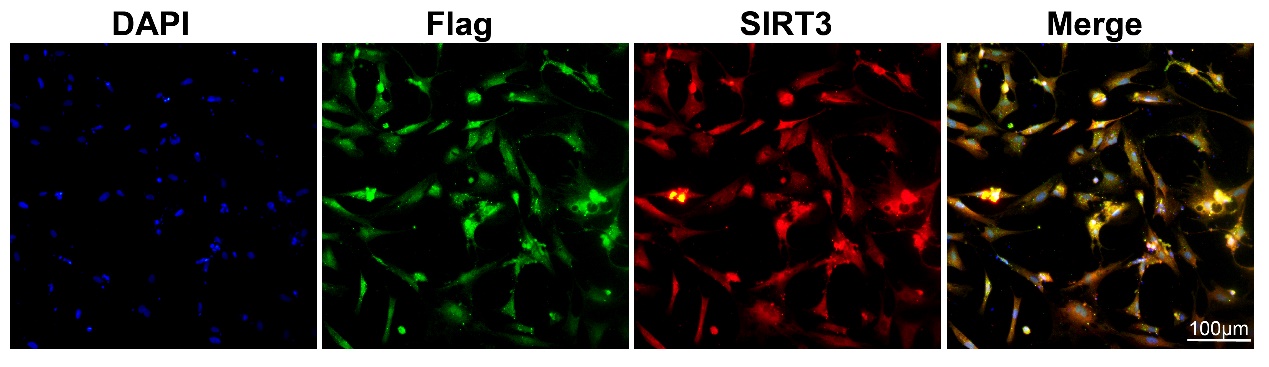


**Figure S3. Overexpression efficiency of SIRT3 in NRCMs.** Representative immunofluorescence staining images of SIRT3 and Flag in NRCMs.
