# Supplementary figures and images for: SIRT3 regulates cardiolipin biosynthesis in pressure overload-induced cardiac remodeling by PPARγ-mediated mechanism
Source: PLoS One. 2024 Apr 16;19(4):e0301990. doi: 10.1371/journal.pone.0301990 (PMC11020683; doi:10.1371/journal.pone.0301990)

Fig. 2E

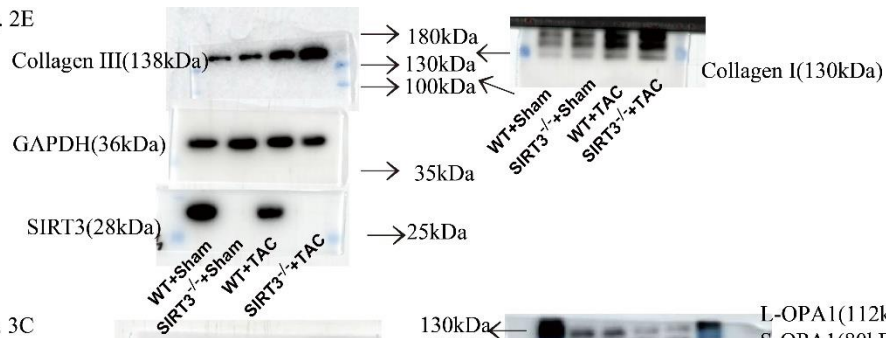

Fig. 3C

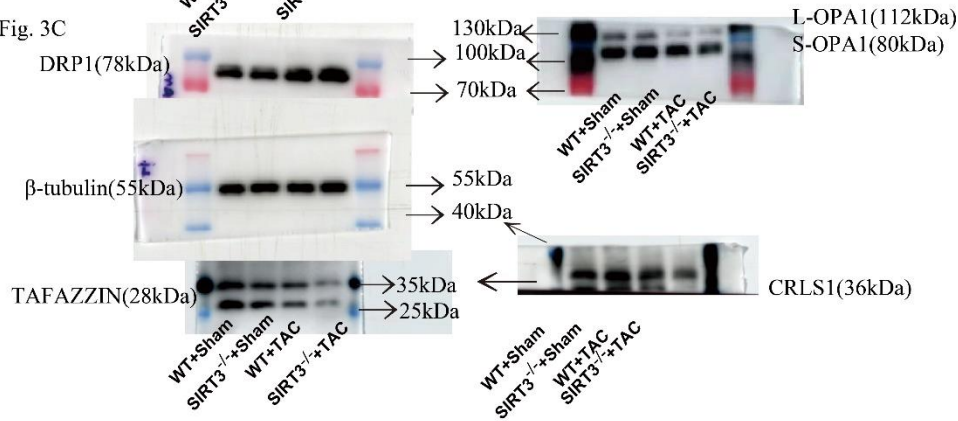

Fig. 5M

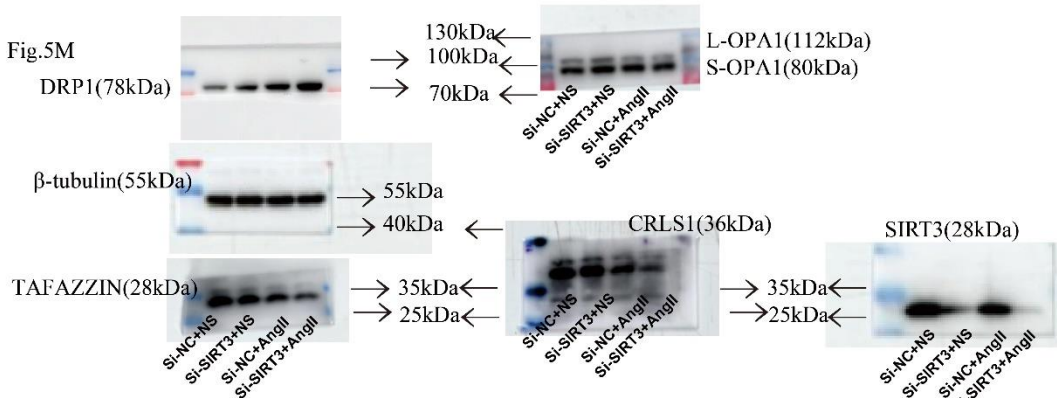

Fig. 5S

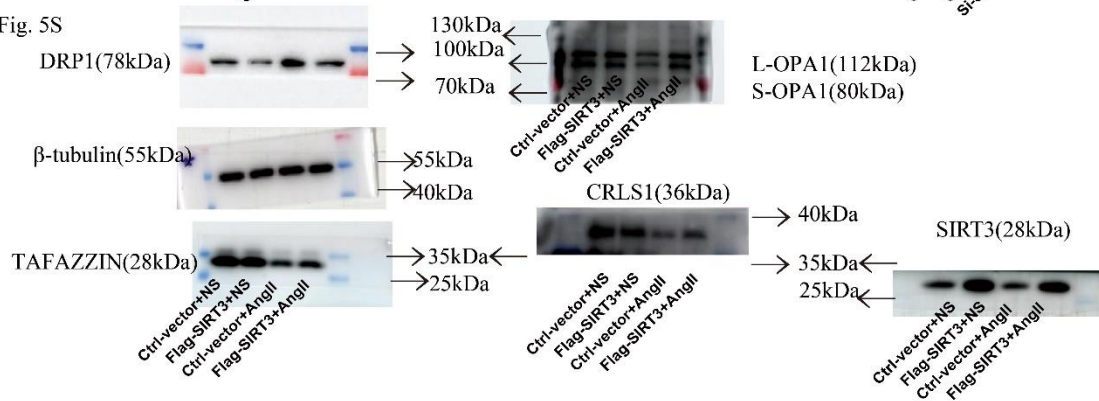

Figure 6A

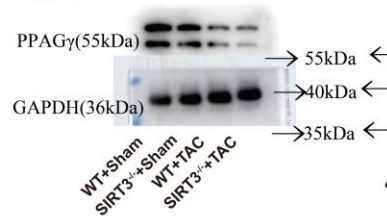

Figure 6B

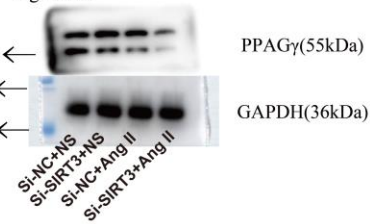

Figure 6C

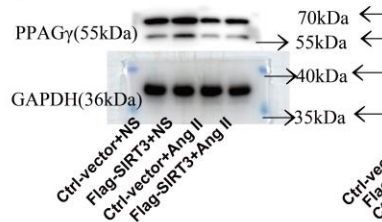

Figure 6D

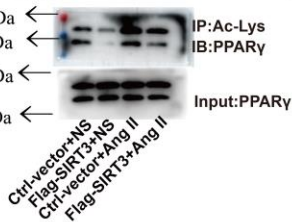

Figure 6I

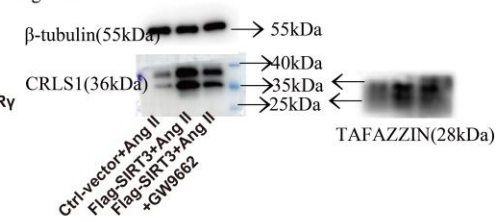

Figure S2A

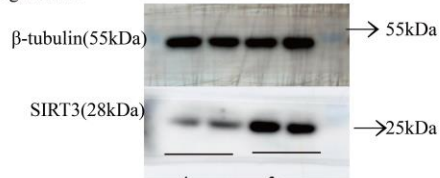

Figure S2D

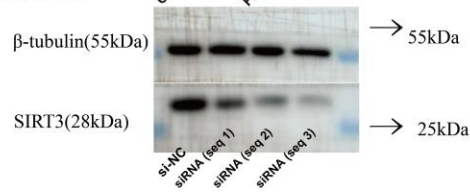

Supplement: S1 Raw images — (PDF) [file pone.0301990.s003.pdf]
